# Sample size and statistical power considerations in high-dimensionality data settings: a comparative study of classification algorithms

**DOI:** 10.1186/1471-2105-11-447

**Published:** 2010-09-03

**Authors:** Yu Guo, Armin Graber, Robert N McBurney, Raji Balasubramanian

**Affiliations:** 1 BG Medicine, Inc., 610 Lincoln St., Waltham, MA 02451, USA; 2 Institute for Bioinformatics and Translational Research, UMIT, Eduard Wallnoefer Zentrum 1, 6060 Hall in Tyrol, Austria; 3 Optimal Medicine Ltd., Warwick Enterprise Park, Wellesbourne, Warwick CV35 9EF, UK; 4 Division of Biostatistics and Epidemiology, University of Massachusetts - Amherst, 715 North Pleasant Street, Amherst, MA 01003, USA

## Abstract

**Background:**

Data generated using 'omics' technologies are characterized by high dimensionality, where the number of features measured per subject vastly exceeds the number of subjects in the study. In this paper, we consider issues relevant in the design of biomedical studies in which the goal is the discovery of a subset of features and an associated algorithm that can predict a binary outcome, such as disease status. We compare the performance of four commonly used classifiers (K-Nearest Neighbors, Prediction Analysis for Microarrays, Random Forests and Support Vector Machines) in high-dimensionality data settings. We evaluate the effects of varying levels of signal-to-noise ratio in the dataset, imbalance in class distribution and choice of metric for quantifying performance of the classifier. To guide study design, we present a summary of the key characteristics of 'omics' data profiled in several human or animal model experiments utilizing high-content mass spectrometry and multiplexed immunoassay based techniques.

**Results:**

The analysis of data from seven 'omics' studies revealed that the average magnitude of effect size observed in human studies was markedly lower when compared to that in animal studies. The data measured in human studies were characterized by higher biological variation and the presence of outliers. The results from simulation studies indicated that the classifier Prediction Analysis for Microarrays (PAM) had the highest power when the class conditional feature distributions were Gaussian and outcome distributions were balanced. Random Forests was optimal when feature distributions were skewed and when class distributions were unbalanced. We provide a free open-source R statistical software library (*MVpower*) that implements the simulation strategy proposed in this paper.

**Conclusion:**

No single classifier had optimal performance under all settings. Simulation studies provide useful guidance for the design of biomedical studies involving high-dimensionality data.

## Background

High-content experiments utilizing 'omics' technologies are increasingly being conducted to discover molecular biomarkers that discriminate between two or more phenotypic classes of interest. Recent examples of applications include a study to identify differential gene expression patterns to distinguish different sub-classes of pediatric and adult leukemia[1] and a proteomic study to detect serum based biomarkers for the diagnosis of head and neck cancers[2]. Such experiments typically involve measurements of thousands of biomolecular features from each subject in the study - however, the vast majority of the measured entities do not exhibit differences in mean intensity levels between the different classes of comparison. The goal of these studies is to identify the subset of features or 'biomarker set' that is associated with class membership and a corresponding algorithm that can predict class membership with sufficiently high accuracy. Thus, computational methods employed in the analysis of such experiments often involve a two-stage process: the first involving a dimensionality reduction procedure for identifying the subset of features that is significantly associated with class membership and the second to estimate a class prediction algorithm based on the selected subset of features that can be used to predict a subject's class.

Efforts to estimate statistical power and sample size requirements in the design of high-content experiments must take into account the data analytic procedures employed to reduce dimensionality that precede the estimation of the optimal classification rule. Several classifiers are commonly used in the analysis of 'omics' data, including Random Forests[3], Prediction Analysis for Microarrays[4], K-nearest neighbor classification[5] and Support Vector Machines[6]. Each of these classifiers involves complex algorithms based on a variety of assumptions, thus their relative performance is naturally expected to vary depending on the application and the nature of the data.

Several methods have been reported to estimate sample size and statistical power for detecting individual features significantly associated with phenotypic class, while adjusting for multiple comparisons and false discovery rate in high-dimensional data[7-16]. Similarly, comparison of the performance of multivariate classifiers has increasingly become an active area of research. Huang et al. (2002)[17] proposed minimum sample size requirements based on a Fisher Discriminant Analysis (FDA) procedure when carried out after a subset of features had been selected based on individual F tests. This approach does not account for the variability induced in analysis due to the feature reduction process and focuses on a specific classifier (FDA). Dobbin, K. et al. (2007)[18] presented analytical solutions for statistical power and sample size based on parametric assumptions with regard to feature distributions. The method estimates the minimum sample size required such that the expected probability of correct classification of the resulting predictive function is within a specified threshold of the best achievable probability of correct classification. The algorithm presented in Dobbin, K. et al. (2007)[18] is valuable in that it addresses issues related to the high-dimensionality of the data and the variability introduced during both the feature reduction and classifier training procedures - however, the method does not provide guidance on the relative performance of classification algorithms. Aliferis C.F., et al. (2009)[19] presented results from simulation studies comparing two protocols for feature selection and classifier training - the first protocol involved feature selection using Pearson's Correlation in conjunction with a Nearest-Centroid Prediction method; the second protocol was based on the Support Vector Machine algorithm without a preceding feature reduction procedure. Hua J., et al. (2009)[20] presented an extensive simulation study comparing the performance of several feature reduction procedures in conjunction with three classifiers (namely, Linear Discriminant Analysis, Nearest Neighbors and Support Vector Machines). The authors compared the various methods with respect to the highest achievable classification accuracy of the resulting classification algorithms. The extensive simulation studies presented in Hua J., et al. (2009)[20] shed light on the relative performance of several feature reduction strategies under diverse data settings. However, this study does not provide guidance on the optimal sample size needed to achieve a desired level of statistical power, when multivariate classifiers are used in conjunction with dimensionality reduction procedures.

The objective of this paper is to present a comparative study of the performance of four commonly used classifiers, namely Random Forests[3], Prediction Analysis for Microarrays[4], K-nearest neighbor classification[5] and Support Vector Machines[6]. The statistical power of each of these algorithms is evaluated in high-dimensionality data settings where the analysis includes a recursive feature elimination procedure to identify the subset of features that are significantly associated with class membership. We compare the performance of each classifier under varying sample size and levels of signal-to-noise in the dataset. We also assess the effect of non-Gaussian (skewed) feature distributions, correlation between biomarkers, imbalance in class distribution and the choice of metric for quantifying classifier performance. We also provide a free open-source R statistical software[21] library (MVpower) that implements the methods proposed in the paper.

To guide the design of 'omics' studies, we present a summary of the key characteristics of metabolite and proteomic data profiled in seven experiments utilizing high-content mass spectrometry and multiplexed immunoassay-based techniques. The experiments undertaken at BG Medicine Inc.[22] in collaboration with other institutions span a wide variety of disease settings including organ toxicity, cardiovascular disease, infectious disease, and neuromuscular disease. Since statistical power is fundamentally dependent on the difference in mean intensity levels between the phenotypic classes being compared, we provide summaries of effect sizes observed in the data profiled in these experiments. We define effect size as the absolute difference in mean intensity of each feature between two phenotypic classes, relative to the average within-class standard deviation. We also present data on the percentages of features in each study with significantly non-Gaussian class conditional feature distributions.

In the settings considered in this paper, we assume that the subjects in a typical study belong to one of two mutually exclusive classes representing distinct phenotypic groups of interest (e.g. diseased versus healthy) - the two classes are referred to as 'cases' and 'controls', respectively. The biomolecular entities profiled on each subject using mass spectrometry and multiplexed immunoassay based proteomic and metabolomic technologies are referred to as 'features'. The subset of features with non-zero difference in mean intensity, between cases and controls are referred to as 'biomarkers'. The subset of features with zero difference in mean intensity between cases and controls are referred to as 'noise features'. Aliquots of body fluid (e.g. plasma, serum) or tissue from each subject from which metabolite and protein measurements are made are referred to as 'samples'.

## Results

This section is organized as follows: In the first sub-section ('Data') we present a summary of the key characteristics of data obtained from high-content 'omics' experiments from several studies involving human subjects or animal models. The data summaries from several 'omics' studies are presented to guide selection of parameters and models relevant in power calculations for future investigations involving similar high-throughput methods.

In the second sub-section ('Simulation Results'), we present results from simulation studies comparing the performance of classifiers K-nearest neighbor (KNN), Prediction Analysis of Microarrays (PAM), Random Forests (RF) and Support Vector Machines (SVM) in high-dimensionality data settings.

### Data

In Table 1, we present a brief description of seven high-content experiments, including information on the primary disease area, species, samples analyzed and the specific platforms employed for profiling proteins and metabolites. In all the experiments considered, the primary outcome was dichotomous, representing two mutually exclusive phenotypic classes of interest. A brief description of each study is provided in the Methods Section.

**Table 1:** Summary of data arising from experiments utilizing 'omics' technologies.

Study (Compartment)	I (Plasma)	II (Plasma)	III (Plasma)	IV (Plasma)	V (Tissue)	VIa (Tissue)	VIb (Plasma)	VIIa (Tissue)	VIIb (Plasma)
Disease area	Organ Toxicity	Cardio-vascular Disease	Infectious Disease	Neuro-muscular Disease	Organ Toxicity	Organ Toxicity		Organ Toxicity	
**Species**	Human	Human	Mouse	Mouse	Rat	Rat		Rat	
Platforms									
- GC/MS	√	√	√				√		
- Lipid LC/MS	√	√	√	√	√	√	√	√	√
- Polar LC/MS	√	√	√	√	√	√	√	√	√
- Proteomics (Mass Spectrometry)	√	√	√	√	√			√	√
- Proteomics (Multiplexed Immunoassay)		√							√

The metabolomic platforms utilized in various studies include GC/MS (gas chromatography coupled with mass spectrometry), Lipid LC/MS (liquid chromatography coupled with mass spectrometry platform targeting lipids) and Polar LC/MS (liquid chromatography coupled with mass spectrometry platform targeting polar metabolites).

Comprehensive metabolite profiling of the individual samples was based on a combination of three platforms employing mass spectrometry (MS) based techniques referred to as GC/MS, Lipid LC/MS and Polar LC/MS. Proteomic analysis was based on a combination of targeted methods using quantitative multiplexed immunoassay technique as well as a comprehensive protein profiling strategy based on tandem mass spectrometry. A brief description of the platforms and the specific molecules targeted by each is provided in the Methods Section.

**Figure 1:**
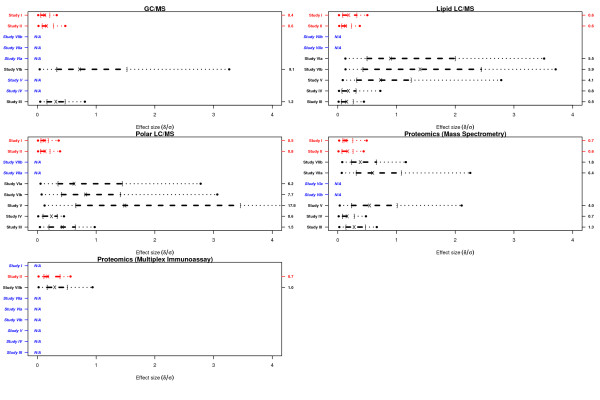
**Distribution of effect size among features measured in each platform per study**. Solid dots indicate 5^th ^and 95^th ^percentiles, brackets indicate 1^st ^and 3^rd ^quartiles, × indicates the median value and the number noted on the right axis indicates maximum value of effect size. Studies of human (animal model) samples are colored red (black). Platforms that were not utilized for a specific study are denoted as N/A (blue).

**Figure 2:**
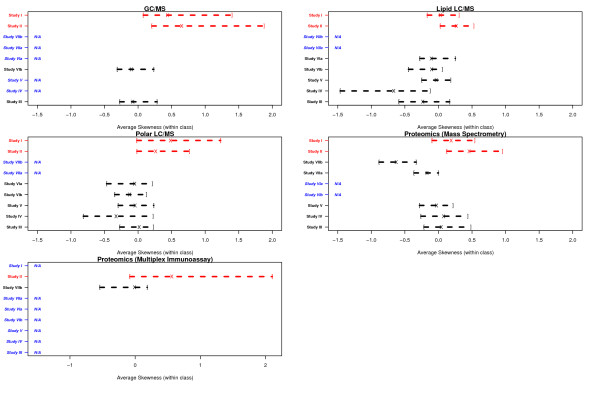
**Distribution of skewness among features measured in each platform per study**. Brackets indicate 1^st ^and 3^rd ^quartiles and × indicates the median value. Studies of human (animal model) samples are colored red (black). Platforms that were not utilized for a specific study are denoted as N/A (blue).

**Figure 3:**
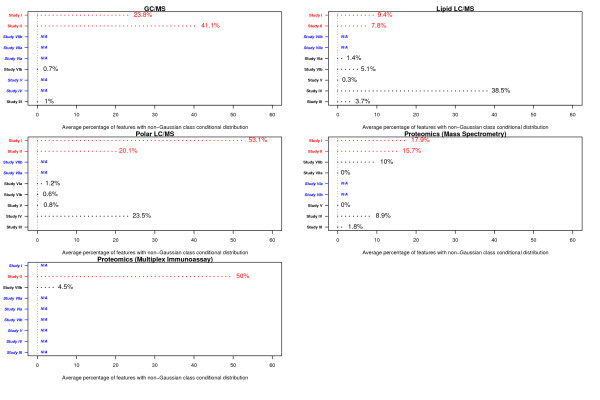
**Percentage of analytes with significantly non-Gaussian distributions, in each platform per study**. Studies of human (animal model) samples are colored red (black). Platforms that were not utilized for a specific study are denoted as N/A (blue).

Feature intensity measurements were transformed to the natural logarithm scale prior to data analysis. Datasets generated by individual platforms in each study were summarized with regard to the following: (i) distribution of effect sizes, (ii) distribution of skewness and (iii) the percentage of features with non-Gaussian distributions (Figures 1, 2, 3).

For each feature (on the natural logarithm scale), we define effect size as follows: Let μ_j _and σ_j _denote the mean and standard deviation within class *j*, respectively, for *j *= 1, 2. Let δ = |μ_1 _- μ_2_| denote the absolute difference in means between the two classes. Assuming that the standard deviations in both classes are equal (denoted by σ), the effect size was defined as the ratio $\frac{\delta }{\sigma }$. The assumption of a common standard deviation within different phenotypic classes was largely borne out in the 'omics' experiments summarized here (details available upon request).

Information on the distribution of skewness of features (on the natural logarithm scale) and the percentage exhibiting significant non-Gaussian distributions reveal important characteristics of the profiled data in each platform/study. For each feature, *x*, skewness was estimated as the ratio $\frac{\sum_{i=1}^{n}{\left(x_{i}-\mu \right)}^{3}}{\sigma^{3}}$, where *n *denotes the number of subjects within a class, *x_1_,.., x_n _*denote the *n *intensity measurements for that feature, μ represents the mean and σ denotes the standard deviation for that feature. For each feature, skewness was estimated separately within each case and control class - the distribution of the average of the within class estimates is reported in Figure 2. Lastly, we estimated the percentage of features within each dataset (per platform per study) that deviated significantly from the assumption of a class conditional Gaussian distribution. For each feature, we obtained a *p *value from the Anderson-Darling test for normality[23] and adjusted for multiple comparisons using the *q *value procedure[24]. In each phenotypic class, the percentage of features with *p *and *q *values less than 0.05 was estimated - the average of the class-specific percentages is reported in Figure 3.

In Figure 1, we present the distribution of effect sizes for features (on natural logarithm scale) measured in each 'omics' platform per study. Studies on human samples (shown in red) exhibit distributions shifted to the left toward lower effect sizes in all platforms when compared to studies conducted on animal models (shown in black). The maximum effect size in human studies ranged from 0.4 (Study I, GC/MS) to 0.8 (Study II, Polar LC/MS), with the 95^th ^percentile at or below 0.8 in all platforms. The range of effect sizes observed in studies involving animal models (Study III - Study VII, Table) was dramatically larger than that in human studies - the maximum effect size ranged from 0.6 (Study III, Lipid LC/MS platform) to 17.8 (Study V, Polar LC/MS platform). The 95^th ^percentile of the distribution of effect sizes in animal studies was lower than 2.5 in both proteomic platforms. On the other hand, the 95^th ^percentile of the distribution of effect sizes in animal studies among platforms profiling metabolites ranged from approximately 0.4 (Study III, Lipid LC/MS) to larger than 4.0 (Study V, Polar LC/MS). Differences in the magnitude of effect sizes observed between animal and human studies can be attributed in part to the following factors: (i) the increased genetic homogeneity in the animal populations when compared to human populations, and (ii) the nature of the studies undertaken. Animal studies involve in-bred and genetically homogenous populations, thus resulting in reduced within class variability in measured features when compared to studies involving human subjects. Moreover, the animal studies reported here involved experiments comparing the effects of treatment with drug relative to placebo, whereas the human studies were case-control experiments comparing subjects belonging to two distinct disease-related outcome classes.

In Figure 2, we present the distribution of skewness of features measured after natural logarithm transformation of the measurements. For each study, we report the distribution of the average within-class estimate of skewness. The average skewness of features measured in human studies (shown in red) was distinctly larger when compared to studies involving animal models (shown in black). The median skewness in human studies ranged from approximately 0.1 (Study I, Lipid LC/MS) to approximately 0.8 (Study II, GC/MS). Among animal studies, the median skewness was approximately 0 in most platforms. Notable exceptions were the Polar LC/MS and Lipid LC/MS data profiled in Study IV as well as the Proteomics (Mass Spectrometry) data profiled in Study VIIb, where the median skewness was approximately -0.5.

In Figure 3, we present the average percentage of features (per platform and study) with a *p *value and *q *value less than 0.05 resulting from the Anderson-Darling test for normality on the natural logarithm transformed data. The percentage of features satisfying the *p *and *q *value thresholds was estimated within each phenotypic class - the average of the within class estimates are reported. Consistent with the data reported in Figure 2, studies involving human subjects (shown in red) resulted in larger percentages of features with significantly non-Gaussian feature distributions, when compared to studies involving animal models (shown in black). Among human studies, the percentage of features with non-Gaussian distributions ranged from 7.8% (Study II, Lipid LC/MS) to 53.1% [Study I, Polar LC/MS]. Among studies involving animal models, the percentage of features with non-Gaussian distributions ranged from 0% [Studies V, VIIa, Proteomics (Mass Spectrometry)] to 38.5% (Study IV, Lipid LC/MS). The Lipid LC/MS and Polar LC/MS platforms in Study IV were observed to be exceptions among studies involving animal models - in both platforms, significant percentages of features with negative skewness were observed (Figure 2), with correspondingly large percentages of features exhibiting non-Gaussian distributions (Figure 3).

The large average skewness (Figure 2) and the correspondingly high percentage of features exhibiting non-Gaussian distributions (Figure 3) in human studies is consistent with previous reports of larger biological variability and the higher likelihood of observing outlier measurements in human studies, when compared to studies involving animal models[25].

### Simulation Results

We conducted simulation studies comparing the performance of the classifiers KNN, PAM, RF and SVM, in settings in which the number of features exceeded the number of subjects in the study. Each classifier incorporated a recursive feature elimination procedure to select the subset of features that maximized the classifier's performance in predicting class membership. In the following, the sample size in each class is denoted as *n *and the percentage of biomarkers among 1000 features measured per subject is denoted as *k*. References to effect size pertain to individual features comprising the biomarker set - for example, a simulation setting described as '*k *= 1% and effect size of 0.2' refers to a set of 10 biomarkers, each with effect size of 0.2.

The results reported in this paper are based on 100 simulated datasets. The simulations assumed a range of values for *n *(50, 100, 150, 200) and *k *(0.5%, 1%, 5%). The performance of each classifier was quantified based on two metrics: 'average classification accuracy' (i.e. average percentage of samples that were correctly classified) or the 'AUC statistic' associated with the estimated Receiver Operating Characteristics (ROC) curve. The aforementioned metrics to quantify classifier performance were estimated based on samples left out of the classifier training procedure, during a 4-fold cross validation process. Statistical power to detect a 'biomarker set' and associated classification rule was estimated by comparing the observed value of the classifier performance metric to its distribution under the null hypothesis. The null distribution was estimated based on 100 simulated datasets in which 100% of the features measured were noise features. Statistical power was estimated as the percentage of simulated datasets in which the observed value of classifier performance metric exceeded the 95^th ^percentile of its distribution obtained under the null hypothesis. Details on the simulation studies are presented in the Methods section.

#### Presence of noise features

**Figure 4:**
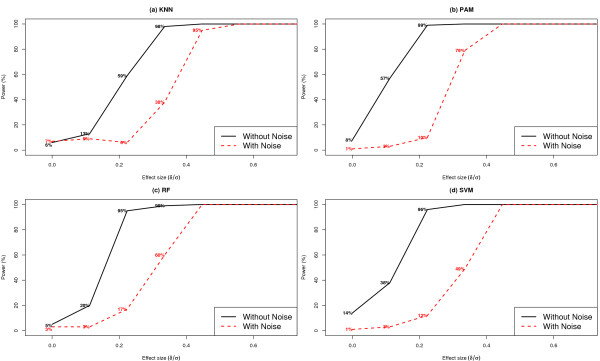
**Statistical power of classifiers KNN, PAM, RF and SVM, comparing settings with and without noise**. Each dataset included *n *= 150 subjects per class, where features were distributed according to a Gaussian distribution within each class. The setting "With Noise" corresponded to the inclusion of 1000 features per subject, where only 10 were biomarkers. The setting "Without Noise" corresponded to the inclusion of only 10 biomarkers per subject. Results are based on 100 simulated datasets.

In Figure 4, we present simulation results illustrating the effect of the presence of noise features on statistical power of each classifier. We compared the setting in which the dataset included only 10 biomarkers per subject ('Without Noise') to the setting in which the dataset included 1000 features per subject, of which 10 were biomarkers ('With Noise'). We assumed that *n *= 150 and that the class conditional feature distributions follow a Gaussian model. In the setting 'With Noise', the optimal subset of features was determined by recursive feature elimination (See Methods). The simulations used average classification accuracy as the metric to quantify classifier performance. The introduction of noise features and the subsequent procedure to determine the biomarker set resulted in drastic reduction in power for effect sizes in the range 0.20 - 0.45, for all classification algorithms considered (Figure 4). For example, when each of the 10 biomarkers was characterized by an effect size of 0.35, statistical power decreased from 100% in the absence of noise features to 38% [95% CI: (28%, 48%)], 79% [95% CI: (71%, 87%)], 60% [95% CI: (50%, 70%)] and 49% [95% CI: (39%, 59%)] in the presence of noise, for KNN, PAM, RF and SVM, respectively. PAM achieved the highest power in the presence of noise when compared to KNN, RF and SVM in this setting. For effect sizes greater than 0.45, all classifiers achieved power approaching 100%, even in the presence of a significant percentage of noise features.

#### Comparison of classification algorithms when class conditional feature distributions are Gaussian

We compared the statistical power of classifiers KNN, PAM, RF and SVM, when the feature distributions in the case and control classes were assumed to follow a Gaussian model. The simulations compared the effects of varying levels of signal-to-noise in the dataset, varying sample sizes, imbalance in class distribution and the choice of metric to measure classifier performance (average classification accuracy Vs AUC).

We first consider the Gaussian distribution to model feature distributions within class as this is a common assumption made in several statistical algorithms, including PAM. In many practical applications, the assumption of a Gaussian distribution may be approximately satisfied by suitable transformations of the data, such as the commonly used logarithm transformation. However, as seen in Figures 2 and 3, several protein and metabolite features particularly from human studies exhibit highly skewed, non-Gaussian distributions. Additionally, data from 'omics' studies indicated that: (i) both biomarkers and noise features are equally likely to have highly skewed distributions within each class; and, (ii) the class conditional feature distributions do not vary significantly by class for most features. Subsequent simulations evaluated the performance of KNN, PAM, RF and SVM when the class conditional feature distributions were modeled according to a mixture of Gaussian distributions.

**Figure 5:**
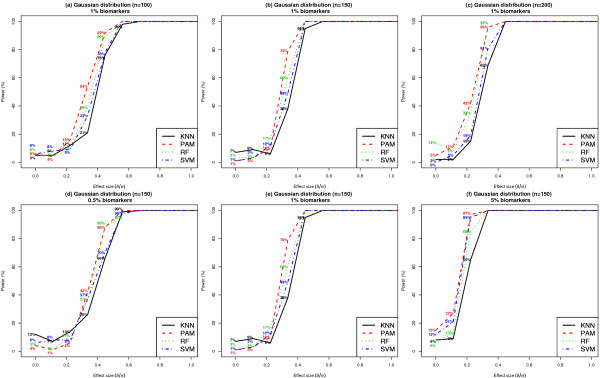
**Comparison of statistical power of classifiers when feature distributions within class are Gaussian**. Each dataset included 1000 features per subject, where features were distributed according to a Gaussian distribution within each class. Results shown in Panels (a) - (c) were based on ***k ***= 1% and ***n ***= 100, 150 or 200. Results shown in Panels (d) - (f) were based on ***n ***= 150 and ***k ***= 0.5%, 1% or 5%. Results are based on 100 simulated datasets. See Additional File 1: Supplemental Figure S1 (a) for similar results when ***n ***= 50 and ***k ***= 1%.

Figure 5 panels (a) - (c) present results on statistical power comparing settings of varying values of *n *(100 Vs 150 Vs 200). Similar results on statistical power for *n *= 50 can be found in Additional File 1: Supplemental Figure S1 (a). Simulations assumed that *k *= 1% and that the class conditional feature distributions follow a Gaussian model. The results are based on the use of average classification accuracy as the metric to quantify classifier performance. When *n *= 100 (150, 200), all four classifiers achieved nearly perfect power (100%) corresponding to effect sizes in individual biomarkers of 0.56 (0.45, 0.45) or greater. For sample sizes of at most *n *= 150 and effect sizes lower than 0.45, PAM achieved the highest power, followed by RF, SVM and KNN. For example, when the effect size of each individual biomarker was 0.34 and *n *= 100, statistical power was 21% [95% CI: (13%, 29%)], 54% [95% CI: (44%, 64%)], 39% [95% CI: (29%, 49%)] and 33% [95% CI: (24%, 42%)], for KNN, PAM, RF and SVM, respectively. Similarly, when *n *= 150 and effect size was 0.34, statistical power was highest for PAM (79% [95% CI: (71%, 87%)]), followed by RF (60% [95% CI: (50%, 70%)]), SVM (49% [95% CI: (39%, 59%)]) and KNN (38% [95% CI: (28%, 48%)]).

Figure 5, panels (d) - (e) present results on statistical power comparing settings of varying values of *k *(0.5% Vs 1% Vs 5%). Simulations assumed that *n *= 150 and that the class conditional feature distribution follows a Gaussian model. All four classifiers achieved nearly perfect power (100%) to detect effect sizes of 0.56 (0.45, 0.34) or greater, when *k *= 0.5% (1%, 5%). When *k *= 0.5% and the effect size of each individual biomarker was 0.34, statistical power was 26% [95% CI: (17%, 35%)], 42% [95% CI: (32%, 52%)], 37% [95% CI: (28%, 46%)] and 37% [95% CI: (28%, 46%)], for KNN, PAM, RF and SVM, respectively. When *k *= 1% and the effect size of each individual biomarker was 0.34, statistical power was 38% [95% CI: (28%, 48%)], 79% [95% CI: (71%, 87%)], 60% [95% CI: (50%, 70%)] and 49% [95% CI: (39%, 59%)], for KNN, PAM, RF and SVM, respectively. For modest effect sizes, PAM achieved the highest power, followed by RF, SVM and KNN.

#### Comparison of simulation results with methods described in Dobbin et al. (2007)[18]

**Figure 6:**
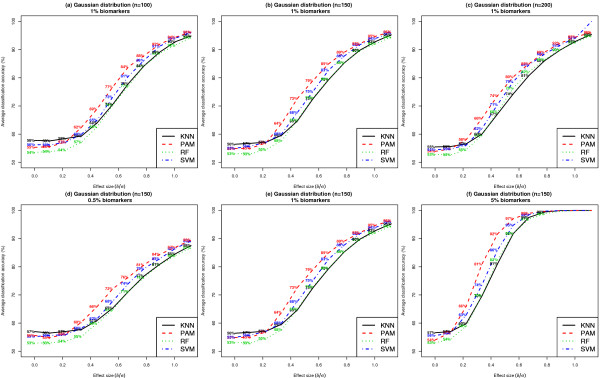
**Comparison of average classification accuracy of classifiers when feature distributions within class are Gaussian**. Each dataset included 1000 features per subject, where features were distributed according to a Gaussian distribution within each class. Results shown in Panels (a) - (c) were based on ***k ***= 1% and ***n ***= 100, 150 or 200. Results shown in Panels (d) - (f) were based on ***n ***= 150 and ***k ***= 0.5%, 1% or 5%. Results are based on 100 simulated datasets. Average classification accuracy estimates were derived based on a 4-fold cross validation procedure. See Additional File 1: Supplemental Figure S2 (a) for similar results when ***n ***= 50 and ***k ***= 1%.

Figure 6 presents the average classification accuracy for each classifier, under the assumption that the class conditional feature distributions are Gaussian. The value of *k *was varied between 0.5%, 1% and 5% and *n *was varied between 100, 150 and 200. Similar results on average classification accuracy for *n *= 50 can be found in Additional File 1: Supplemental Figure S2 (a). The estimates of average classification accuracy were based on predictions in samples held out of the classifier training process during a 4-fold cross validation procedure. Following the trend in results shown in Figure 5, PAM achieved the highest average classification accuracy when effect sizes for individual biomarkers was at least 0.34. Assuming *n *= 150, *k *= 1% and effect size of 0.34, the expected percentage of correct classification determined using the algorithm proposed by Dobbin et al. (2007)[18] was 60%. For the same setting, the simulation estimates of average classification accuracy for the classifiers KNN, PAM, RF and SVM were 60% [95% CI: (50%, 70%)], 64% [95% CI: (55%, 73%)], 58% [95% CI: (48%, 68%)] and 60% [95% CI: (50%, 70%)], respectively. The simulation based estimates of average classification accuracy showed close agreement with those derived using Dobbin et al. (2007). In addition, the simulations revealed differences between classifiers with regard to the variance of the average classification accuracy estimates, thereby resulting in significant differences in statistical power between classifiers (Figure 5). In all settings considered in which the features followed a Gaussian class conditional feature distribution, PAM outperformed KNN, RF and SVM.

To address concerns regarding the validity of cross-validation based estimates of average classification accuracy in small samples, an additional alternative strategy was considered. In this procedure, the classifiers were trained following a recursive-feature elimination procedure incorporating a 4-fold cross validation process as described in the Methods. However, the average classification accuracy of each estimated algorithm was assessed based on an independent test set of 400 subjects (See Additional File 1: Supplemental Figure S3 (a)). The training datasets were simulated following a Gaussian model, where *n *= 150 and *k *= 1%. For an effect size of 0.34, the independent test set based estimates of average classification accuracy for the classifiers KNN, PAM, RF and SVM were 57% [95% CI: (47%, 67%)], 60% [95% CI: (50%, 70%)], 56% [95% CI: (46%, 66%)] and 58% [95% CI: (48%, 68%)], respectively. In the settings considered in these simulations, the estimates of average classification accuracy based on independent test sets were similar to those obtained using the 4-fold cross validation procedure.

#### Correlated features

**Figure 7:**
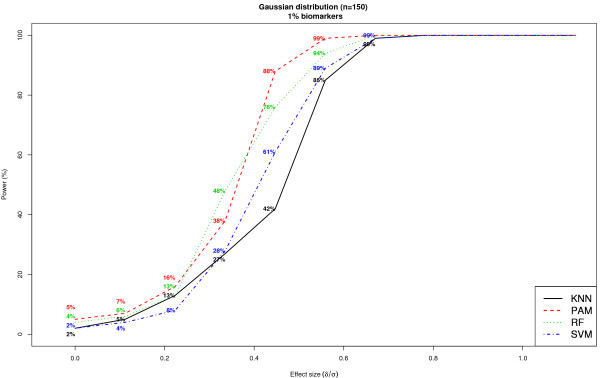
**Comparison of statistical power of classifiers when feature distributions within class are Gaussian and the biomarkers are correlated**. Each dataset included 1000 features per subject, where features were distributed according to a Gaussian distribution within each class. The results are based on ***k ***= 1% and ***n ***= 150. In order to simulate dependence between biomarkers, the data generation model assumed a correlation of 0.5 between the 10 biomarkers in both cases and controls. Results are based on 100 simulated datasets.

In the previous simulations, we assumed that the distributions of the 1000 features measured per subject were statistically independent. However, this assumption breaks down in several applications due to the inherent dependence of biomarkers that belong to similarly acting biological pathways. To model this dependence between biomarkers, the previously described Gaussian data generation model was generalized to incorporate a correlation of 0.5 between the 10 biomarkers in both the cases and controls. The 990 noise features were assumed to be independent in both cases and controls. The results from this simulation are shown in Figure 7. When *k *= 1% and the effect size of each individual biomarker was 0.34, power was 27% [95% CI: (18%, 36%)], 38% [95% CI: (28%, 48%)], 48% [95% CI: (38%, 58%)] and 28% [95% CI: (19%, 37%)], for KNN, PAM, RF and SVM, respectively. As expected, when the biomarkers were correlated, the resulting statistical power was lower among all classifiers when compared to the setting in which all features were independent (Figure 5b). In this setting of correlated features, RF and PAM resulted in approximately similar power for modest effect sizes between 0.34 and 0.56 and outperformed both KNN and SVM.

#### Imbalance in class distribution

**Figure 8:**
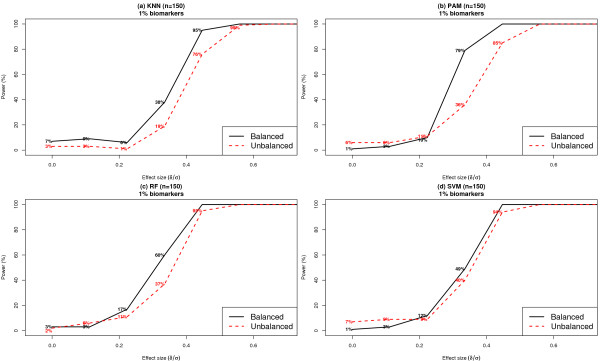
**Comparison of statistical power of classifiers under balanced versus unbalanced class distributions**. Each dataset included 1000 features per subject with ***k ***= 1%, where features were distributed according to a Gaussian distribution within each class. A balanced design including 150 subjects in each class was compared to an unbalanced design of 100 cases and 200 controls. Results are based on 100 simulated datasets.

In many biomedical applications, the sample sizes in the case and control classes tend to be significantly unbalanced. Such situations are common in studies of rare diseases, where an investigator may have limited access to samples from individuals with disease when compared to healthy control subjects. Imbalance in class sizes significantly influences the performance of classifiers and as a consequence, the resulting statistical power can be dramatically affected. In the simulation results depicted in Figure 8, we compared the setting where the dataset included 100 cases and 200 controls to the setting where there were 150 subjects in each class. We further assumed that *k *= 1% and that the class conditional distributions follow a Gaussian model. The simulations used average classification accuracy as the metric to quantify classifier performance. For PAM and RF, the prior class weights were specified to be equal, to adjust for the imbalance in class distributions during the classifier training process. In all four classifiers, the imbalance in class sizes resulted in a significant decline in statistical power for effect sizes lower than 0.45. For example, when the effect size of individual biomarkers was 0.34, statistical power reduced from 38% [95% CI: (28%, 48%)] to 19% [95% CI: (11%, 27%)] for KNN, from 79% [95% CI: (71%, 87%)] to 36% [95% CI: (27%, 45%)] for PAM, from 60% [95% CI: (50%, 70%)] to 37% [95% CI: (28%, 46%)] for RF and from 49% [95% CI: (39%, 59%)] to 40% [95% CI: (30%, 50%)] for SVM, respectively. The greatest and least decline in power due to imbalance in class sizes was observed in PAM and SVM, respectively. As a caveat, we note that the AUC statistic has been previously identified as having limitations in settings of unbalanced outcomes[26-28] - these limitations may also influence the results reported here.

#### Choice of metric to quantify classifier performance

**Figure 9:**
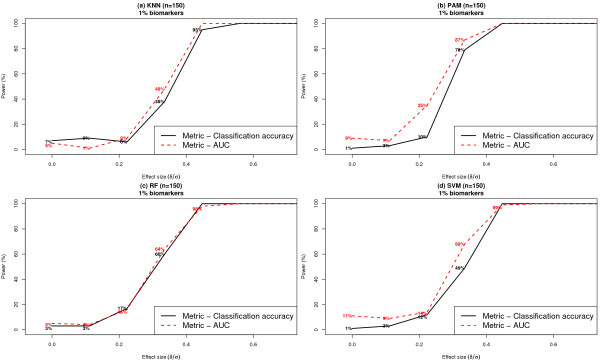
**Comparison of statistical power of classifiers, under different metrics for assessing classifier performance**. Each dataset included 1000 features per subject, where features were distributed according to a Gaussian distribution within each class. The results are based on *k *= 1% and *n *= 150. The classifier performance metric 'average classification accuracy' was compared to the 'AUC statistic', in determining the optimal subset of features after dimensionality reduction. Results are based on 100 simulated datasets.

Figure 9 presents simulation results comparing the effect of the metric used to assess classifier performance on resulting statistical power. Metrics used to quantify performance of a classifier were: (i) average classification accuracy or (ii) the AUC statistic. Average classification accuracy was defined as the percentage of samples correctly classified as a 'case' or 'control', estimated from class predictions made on samples held out of the classifier training process. The AUC statistic refers to the area under the ROC curve corresponding to the trained classifier, where the AUC statistic was also estimated based on class predictions made on held out samples. The metric selected to quantify classification performance drives the selection of the subset of features upon which each is classifier is trained (See Methods).

The results shown in Figure 9 were based on the assumptions of *n *= 150, *k *= 1% and a Gaussian model for class conditional feature distributions. When the effect size of individual biomarkers was 0.44 or greater, all four classifiers achieved perfect power, regardless of the metric used to assess classifier performance. For effect sizes lower than 0.44, classifiers constructed based on optimizing the AUC statistic resulted in modestly higher power when compared to classifiers optimized based on the average classification accuracy. The distinction was least pronounced for RF, where both metrics resulted in nearly identical power. For example, to detect an effect size of 0.34, estimates of statistical power based on average classification accuracy and AUC were 38% [95% CI: (28%, 48%)] and 48% [95% CI: (38%, 58%)] for KNN, 79% [95% CI: (71%, 87%)] and 87% [95% CI: (80%, 94%)] for PAM, 60% [95% CI: (50%, 70%)] and 64% [95% CI: (55%, 73%)] for RF and 49% [95% CI: (39%, 59%)] and 68% [95% CI: (59%, 77%)] for SVM.

#### Effect of skewed class conditional feature distributions

**Figure 10:**
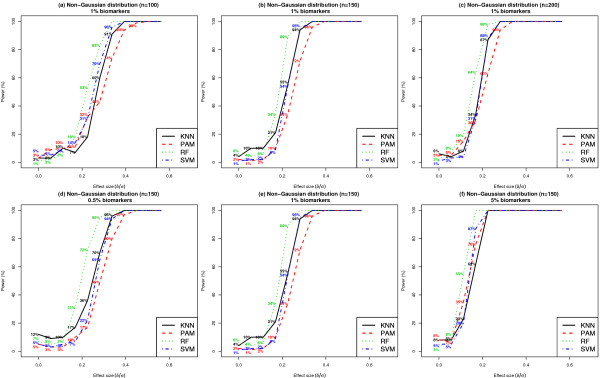
**Comparison of statistical power of classifiers when feature distributions within class are non-Gaussian**. Each dataset included 1000 features per subject, where features were distributed according to a non-Gaussian distribution within each class. Results shown in Panels (a) - (c) were based on ***k ***= 1% and ***n ***= 100, 150 or 200. Results shown in Panels (d) - (f) were based on ***n ***= 150 and ***k ***= 0.5%, 1% or 5%. Results are based on 100 simulated datasets. See Additional File 1: Supplemental Figure S1 (b) for similar results when ***n ***= 50 and ***k ***= 1%.

Figure 10 presents results on statistical power when the class conditional feature distributions followed a non-Gaussian mixture model, for varying values of *n *(100 Vs 150 Vs 200) and *k *(0.5% Vs 1% Vs 5%). Similar results on statistical power when *n *= 50 and *k *= 1% are shown in Additional File 1: Supplemental Figure S1 (b). In all settings compared, RF was the most robust to deviations from the Gaussian model and achieved the highest power when compared to PAM, SVM and KNN. For example, when the effect sizes of individual biomarkers was 0.28, *k *= 1% and *n *= 150, statistical power was 94% [95% CI: (89%, 99%)], 72% [95% CI: (63%, 81%)], 100% and 95% [95% CI: (90%, 100%)], for KNN, PAM, RF and SVM, respectively. When *k *= 1% and *n *= 200, statistical power was 100%, 94% [95% CI: (89%, 99%)], 100% and 100%, for KNN, PAM, RF and SVM, respectively. We note that the parameters chosen to simulate non-Gaussian features resulted in within-class feature variances that were four times as large when compared to that in the Gaussian setting (See Methods). Thus, for features corresponding to a fixed effect size, those in the Gaussian setting had one half the magnitude of mean separation between classes (i.e. |μ_1 _- μ_2_|) when compared to features in the non-Gaussian setting. As a consequence, for a fixed value of effect size, the power achieved by all classifiers was larger in the non-Gaussian (Figure 10) when compared to the Gaussian setting (Figure 5).

In all cases considered, PAM was the least robust to non-Gaussian feature distributions. PAM is a generalization of a Linear Discriminant Analysis classifier, based on the assumption that the features in each class are distributed according to a multivariate normal distribution. Thus, its sensitivity to deviations from a Gaussian model is expected. The non-parametric nature of Random Forests contributes to its robust performance under settings of varying class conditional feature distributions.

**Figure 11:**
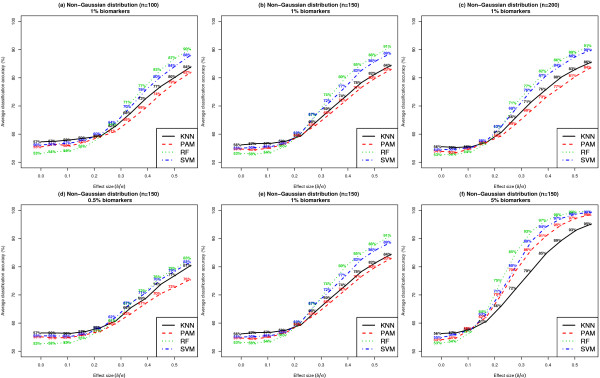
**Comparison of average classification accuracy of classifiers when feature distributions within class are non-Gaussian**. Each dataset included 1000 features per subject, where features were distributed according to a non-Gaussian distribution within each class. Results shown in Panels (a) - (c) were based on ***k ***= 1% and ***n ***= 100, 150 or 200. Results shown in Panels (d) - (f) were based on ***n ***= 150 and ***k ***= 0.5%, 1% or 5%. Results are based on 100 simulated datasets. Average classification accuracy estimates were derived based on a 4-fold cross validation procedure. See Additional File 1: Supplemental Figure S2 (b) for similar results when ***n ***= 50 and ***k ***= 1%.

Figure 11 presents the average classification accuracy for each classifier, under the assumption that the class conditional feature distributions are non-Gaussian. The values of *k *were varied between 0.5%, 1% and 5% and *n *varied between 100,150 and 200. Similar results on average classification accuracy when *n *= 50 and *k *= 1% are shown in Additional File 1: Supplemental Figure S2 (b). Following the trend evident in Figure 10, RF achieved the highest average classification accuracy in all settings considered. To address concerns regarding the validity of cross-validation based estimates in small samples, we pursued an additional alternative strategy to estimate average classification accuracy based on an independent test set of 400 subjects. Additional File 1: Supplemental Figure S3 (b) presents the average classification accuracy estimated using an independent test set. The training datasets were simulated following a non-Gaussian model, where *n *= 150 and *k *= 1%. The estimates of average classification accuracy based on independent test sets were similar to the estimates obtained using the 4-fold cross validation procedure.

#### R package

A free open-source R statistical software library (*MVpower, Version 2.0*) is available for download from the R project website[21]. The R package will allow the user to estimate statistical power based on any of the four classifiers (KNN, PAM, RF and SVM) for user-defined inputs for parameters *n*, *k*, number of features per subject and form of class conditional feature distributions. The R package is made available under the open-source Artistic License 2.0.

## Discussion

The results presented in this paper focus on the minimum sample size required for determining whether a classification algorithm performs significantly better than random chance. Although a specific sample size may result in high power, the resulting classifier may not include all existing biomarkers that truly discriminate between cases and controls. This could also result in biomarker sets that have poor overlap in comparable experiments. The simulation strategy and resulting sample sizes discussed in this paper are appropriate for studies conducted during the initial stage of biomarker discovery - such as clinical settings in which the existence of a biomarker set with clinically useful discriminatory ability between outcome classes is unknown. When the initial phases of investigation have proven to be successful, subsequent validation studies may be designed with more stringent conditions aimed at ensuring the discovery of all relevant biomarker as well as establishing a high degree of reproducibility. As discussed in Ein-Dor et al. (2006)[29], with the added constraint that the discovered biomarker set is sufficiently reproducible, the resulting sample size increases dramatically to the thousands. Such additional constraints to ensure sufficient power to discover a robust biomarker set can be imposed on simulations similar to the ones proposed in this paper for purposes of comparing various classifiers.

The simulation results presented here assumed that the outcome of interest was binary (i.e. case versus control). Further research into sample size requirements for studies with outcomes involving multiple (> 2) phenotypic groups, continuous and or censored measurements (e.g. survival time) is needed.

## Conclusion

In this paper, we presented a summary of the characteristics of 'omics' data profiled in seven experiments involving human subjects or animal models in various disease areas. The average magnitude of effect size observed in studies involving human subjects was markedly lower when compared to that observed in animal studies. Moreover, the datasets arising in human studies were characterized by higher biological variation and the presence of outliers, resulting in skewed, non-Gaussian feature distributions within specific phenotypic classes. The observations of modest effect sizes and larger biological variation greatly influence the sample size needed to adequately power studies in human subjects.

We presented results comparing the performance of four widely used classifiers (KNN, PAM, RF and SVM) in settings where the number of features far exceeds the number of subjects in the study. We illustrated that the presence of high levels of signal-to-noise results in a dramatic reduction in power in all classifiers considered. When the class conditional feature distributions were assumed to follow a Gaussian model, PAM achieved the highest power when compared to KNN, RF and SVM. However, when the feature distributions in each class were non-Gaussian, RF achieved the highest power among all four classifiers. The simulations further illustrated the decrease in power due to imbalance in class sizes - under this scenario, classifiers based on the RF and SVM algorithms resulted in the highest power. The choice of metric to assess performance of a classifier (AUC Versus average classification accuracy) did not result in appreciable differences in statistical power, in the settings considered in this paper.

## Methods

### Data

#### Study I

This was a retrospective case-control study conducted in human subjects to discover diagnostic biomarkers of organ toxicity in human blood plasma samples. The study involved 41 cases and 93 control subjects. The experimental design is described in detail in a previous publication[30].

#### Study II

This was a matched case-control study conducted in human subjects to discover prognostic biomarkers in blood plasma for near-term cardiovascular events. The study involved 68 cases matched to 68 controls. Subjects were matched based on age, gender, race/ethnicity and severity of coronary artery disease.

#### Study III

This was a study conducted in a mouse model of infectious disease, aimed at discovering blood plasma biomarkers predictive of treatment response. The study involved 15 cases and 15 controls.

#### Study IV

This study was conducted in a mouse model of neuromuscular disease to discover blood plasma biomarkers of treatment response. The study involved 46 cases and 22 controls.

#### Study V

This was a study conducted in a mouse model to discover treatment effect biomarkers in organ tissue. The study involved one control (*n *= 12) and two treatment groups (*n *= 12, 17). The distribution of effect size was based on the comparison between the control and the most extreme treatment group.

#### Study VI

This was a study conducted in a mouse model to discover treatment effect biomarkers in blood plasma and organ tissue. The study involved one control (*n *= 14) and four treatment groups (*n *= 11, 12,14,15). The distribution of effect size was based on the comparison between the control and the most extreme treatment group.

#### Study VII

This was a study in a rat model to discover biomarkers of treatment effect in organ tissue (VIIa) and blood plasma (VIIb) samples. The study involved one control (*n *= 12) and six treatment groups (*n *= 12). The distribution of effect size was based on the comparison between the control and the most extreme treatment group. The details regarding the experimental design are provided in a previous publication [31].

### Mass Spectrometry (MS) Platforms

The studies described in this paper employed a combination of one proteomic and three metabolite platforms based on mass spectrometry techniques. The metabolite platforms included a gas chromatography coupled with mass spectrometry technique (GC/MS) targeting specific molecules such as amino acids, sugars, alcohols, aldehydes and cyclohexanols, amines, aromatic compounds, organic acids, phospho-organic acids, sugar acids, sugar amines, sugar phosphates; a liquid chromatography coupled with mass spectrometry platform (Lipid LC/MS) targeting lysophospholipids, phospholipids, cholesterol esters, di- and tri-acylglycerols, sphingomyelins, ceramides and related molecules; and a liquid chromatography coupled with mass spectrometry platform (Polar LC/MS) targeting polar molecules amino acids, amino acid metabolites, organic acids and related molecules. Comprehensive profiling of proteins in the tissue/body fluid was based on protein digestion and chemical isotopic peptide labeling, multi-dimensional liquid chromatography and matrix-assisted laser desorption ionization (MALDI) tandem mass spectrometry analysis of peptides for the identification and quantification of peptides (and proteins). All the mass spectrometry based platforms were carried out at BG Medicine Inc.[22] and TNO Quality of Life[32].

### Proteomic (Multiplex Immunoassay) Platform

Two of the seven studies also included data from the HumanMap (version 1.6) quantitative antigen platform from Rules Based Medicine, Inc.[33]. This multiplexed immunoassay platform detects antibodies to antigens and resulted in quantitative measurements for 89 proteins in each individual sample.

### Bayes Rule

We assume that there are two classes of interest in the population, with equal proportions and that there are *p *biomarkers with non-zero difference in mean intensity between the two classes. Under the assumptions that the class conditional feature distributions follow a multivariate normal distribution, that the covariance matrix of the biomarkers is equal to σ^2^I and that the effect sizes of all biomarkers (δ/σ) are equal, the probability of correct classification is equal to $\Phi (\frac{\delta }{2\sigma }\sqrt{p})$[34].

### Classifiers

We provide brief descriptions of each classifier (KNN, PAM, RF and SVM) below:

**K-nearest neighbor (KNN): **is an intuitive classification method, in which a new subject's class is predicted to be the most frequent class observed among the new subject's '*k*' nearest neighbors[5]. The identity of the nearest neighbors is determined based on a user input measure of distance calculated in the feature space. The algorithm requires the following three inputs (i) a *training data set *including samples with information on features and class membership (ii) parameter '*k*' to determine the number of nearest neighbors considered and (iii) *measure of distance *to be used to determine the nearest neighbors. The key output of the procedure is a predicted class for every subject.

**Prediction Analysis for Microarrays (PAM): **is a classification technique for class prediction that is based on the shrunken centroid technique[4]. Based on a specific value of a threshold (Δ), the shrunken centriods procedure finds a subset of features that are most important in predicting class membership. To implement this procedure, the algorithm calculates the difference between the overall centroid and the class specific centroid for each feature. Features in which the absolute distance between the overall and class specific centroid exceeds Δ in at least one class constitute the shrunken centroids. A new sample is classified to the nearest shrunken centroid. The value of Δ is determined by external cross validation. The algorithm requires as inputs: (i) *a training dataset *including samples with information on features and class and (ii) number of cross validation folds for determining the optimal Δ. Key outputs of the classifier include predicted class membership for each sample and a measure of importance of each feature. For each feature, its measure of importance in classification is proportional to the absolute value of the difference between the overall and class specific shrunken centroids.

**Random Forests**: is a classification procedure based on aggregation of results from several individual classification trees[3]. Each tree in the procedure is grown on a bootstrap sample of the same size as the original dataset. At each node of a tree, a random subset of *m *features is selected. From among the *m *chosen features, the best variable (and its split) based on reduction in Gini impurity is selected. The tree is grown to the fullest extent without pruning. To predict class membership for a subject, their feature values are run down each tree to obtain a predicted class. The predicted class for each sample is then assumed to be the class that obtained the majority vote based on all trees grown. In each tree, since the bootstrap sample used as the training set will approximately exclude 1/3^rd ^of samples, these samples are treated as a 'test set' for assessing prediction error of the classifier. Inputs to the classifier include (i) *a training dataset *including samples with information on features and class labels, (ii) the number of trees to be grown and (iii) number of features (*m*) to be selected at random at each node of every tree. Key outputs of the classifier include predicted class membership for each sample and a measure of variable importance. For each feature, its measure of importance in classification is based on the average decrease in classification accuracy when the feature is randomly permuted when compared to that obtained in the original data.

#### Support Vector Machines

Support Vector Machines (SVM) use kernel functions to transform the original data vector into higher dimensionality spaces. The two phenotypic classes are separated by finding the hyperplane in the transformed feature space which results in the maximal margin of separation between the two classes[6]. The choice of the kernel function (e.g. polynomial, Gaussian) is driven by prior knowledge of the problem. The algorithm requires as input (i) *a training dataset *including samples with information on features and class labels and, (ii) the kernel function. Estimation of the classification accuracy is based on cross validation. The primary output of the algorithm is the predicted class for each sample in the dataset.

### Simulation study

#### Data

Each simulated dataset included measurements on 1000 features per subject, where the sample size per class (*n*) was varied between 50, 100, 150 and 200, respectively. We assumed that subjects belonged to one of two classes, labeled either 'cases' or 'controls'. Unless otherwise stated, each feature was simulated as an independent random variable. A fixed set of *k *percent of the 1000 features were assumed to be differentially expressed in the cases when compared to the controls (referred to as 'biomarkers') and the remaining (*100-k*) percent were assumed to be non-discriminating features between cases and controls (referred to as 'noise'). The percentage of biomarkers (*k*) was varied between 0.5%, 1% and 5%, respectively. The distribution of the features in each class was assumed to follow either a Gaussian distribution or a mixture distribution, respectively. For the simulations in which the features were assumed to follow a Gaussian distribution, the data were generated according to the following:

$$\begin{array}{l} X|'control'\sim N(0,\sigma^{2}=0.20)\\ X|'case'\sim N(\delta ,\sigma^{2}=0.20) \end{array},$$

where δ ranged from 0 to 0.50 in steps of 0.05 for biomarkers. δ was assumed to be 0 for noise features. The simulations in which the features were assumed to follow a non-Gaussian distribution, the data were generated according to the following:

$$\begin{array}{l} X|'control'\sim I[U\leq 0.9]Z_{1}+\text{​}I[U>0.9]Z_{2}\\ X|'case'\sim I[U\leq 0.9]Z_{1}^{*}+\text{​}I[U>0.9]Z_{2}^{*} \end{array},$$

where U, Z_1_, Z_2_, Z_1_* and Z_2_* were assumed to be independent random variables. The distributions of the random variables U, Z_1_, Z_2_, Z_1_* and Z_2_* were:

$$\begin{array}{l} U\sim Uniform(0,1)\\ Z_{1}\sim N(\mu ,\sigma^{2})\\ Z_{2}\sim Uniform(\mu +c_{1}\sigma ,\mu +c_{2}\sigma )\\ Z_{1}^{*}\sim N(\mu^{*},\sigma^{2})\\ Z_{2}^{*}\sim Uniform(\mu^{*}+c_{1}\sigma ,\mu^{*}+c_{2}\sigma ) \end{array}.$$

Here N(μ,σ^2^) refers to a Gaussian distribution with mean μ and variance σ^2^. The following values of the parameters were assumed: σ^2 ^= 0.2, c_1 _= 3.0 and c_2 _= 6.7. Based on the above distributional assumptions, the expected value of each feature (X) among the cases and controls is:

$$\begin{array}{c} E(X|'control')=\mu +\frac{0.1\sigma (c_{2}+c_{1})}{2}\\ E(X|'case')=\mu^{*}+\frac{0.1\sigma (c_{2}+c_{1})}{2} \end{array}.$$

The values of μ and μ* were chosen such that E(X | 'control') = 0 and E(X | 'case') takes the values [0, 0.05, 0.10, ..., 0.50]. Based on the parameters assumed above, the average variance and skewness of each feature (conditional on class) were 0.80 and 1.50, respectively.

#### Recursive Feature Elimination

To find the optimal subset of features, the following recursive feature elimination procedure was carried out for KNN, RF and SVM: An initial classifier was constructed by including all 1000 features as inputs. Subsequent classifiers were constructed based on input datasets that excluded the least important 1% of features in the preceding step. When the input dataset was pruned to 100 or fewer features, the subsequent classifiers excluded the least important feature in each successive feature elimination step. For RF, KNN and SVM, feature importance was estimated based on the variable importance measure output from fitting an RF model - this measure is based on decrease in prediction accuracy when each feature value is randomly permuted. Recursive feature elimination process was carried out by varying the threshold value (Δ) for PAM.

#### Performance Evaluation of Classifier

The average classification accuracy or the AUC statistic was used to assess performance of each classifier. Average classification accuracy was defined as the percentage of samples correctly classified as a 'case' or 'control' based on the predictions made by the classifier. The AUC statistic corresponded to the area under the Receiver Operating Characteristics curve corresponding to the estimated classification algorithm. For each simulated dataset, estimates of average classification accuracy and AUC statistic were based on samples held out of the classifier training process, according to a 4-fold cross validation procedure. That is, corresponding to a single 4-way partition of the samples, 25% of the cases and controls were successively held out of the classifier training process. In each of the 4 steps of the cross validation procedure, a series of classifiers were trained using 75% of the samples, by varying the number of input features according to the recursive feature elimination procedure. The performance of each trained classifier was assessed using either the AUC statistic or average classification accuracy, estimated from class predictions made on 25% of samples that were held out of the classifier training process. For each value of number of features input to the classifier in the training process, the classification performance metric based on either AUC or average classification accuracy was averaged over the results obtained across the 4 cross validation steps. The maximum average value of AUC or classification accuracy among the series of classifiers with varying number of input features (from the recursive feature elimination procedure) was chosen as the measure of classifier performance.

To address concerns regarding the performance of cross-validation based estimates in small samples, we pursued an additional alternative strategy to estimate average classification accuracy. In this modified procedure, the average classification accuracy of each estimated classification algorithm during the recursive feature elimination process was assessed based on an independent test set of 400 subjects. The average classification accuracy estimates based on the cross validation procedure showed close agreement to the estimates derived from the independent test set. Additional File 1: Supplemental Figure S3 presents the average classification accuracy estimated using an independent test set. The training datasets were generated based on *n *= 150 and *k *= 1%.

#### Power Estimation

For each simulated dataset, a *p *value associated with AUC or average classification accuracy was calculated based on comparison of the observed value of the statistic to its distribution under the null hypothesis of no association between the measured features and class label. The null distribution of the AUC statistic and average classification accuracy was obtained from 100 simulated datasets in which all 1000 features were simulated to be non-discriminating between cases and controls. That is, all features were simulated to satisfy E(X | 'control') = E(X |'case') = 0. Statistical power was estimated as the percentage of simulated datasets in which *p *values were at most 0.05. The results reported in the paper are averages based on 100 simulated datasets.

Simulation details specific to each classifier are described below:

#### KNN

Simulations were carried out using the R package *class *(Version 7.2-34)[21]. Each KNN classifier was built by considering the 5 nearest neighbors based on Euclidean distance. In the Recursive Feature Elimination process, feature importance was assessed based the variable importance output obtained from fitting a Random Forests classifier.

#### PAM

Simulations were carried out using the R package *pamr *(Version 1.29)[35]. The Recursive Feature Elimination process was carried out over a vector of 30 threshold values for centroid shrinkage. The maximum value of average classification accuracy or AUC statistic among the 30 classifiers constructed as part of the feature elimination procedure was used as the metric of classification performance. As described above, estimates of average classification accuracy and AUC were based on 'hold out' samples in a 4-fold cross validation procedure.

#### Random Forests

Simulations were carried out using the R package *randomForest *(Version 4.5-18)[21]. In each simulated dataset and for every cross validation partition, a series of Random Forests classifiers consisting of 500 trees each was built, where the number of features randomly selected at each node of a tree (*mtry*) was chosen to equal the square root of the number of input features (rounded up to the nearest integer value). In the Recursive Feature Elimination process, feature importance was assessed based on average decrease in classification accuracy observed resulting from permuting each feature's values[3].

#### SVM

Simulations were carried out using the R package *kernlab *(Version 0.9-7)[21]. Each SVM classifier was built using a Radial Basis kernel ("Gaussian") (*kernel = "rbfdot"*) and bound-constraint classification procedure (*type = "C-bsvc"*). The sigma hyperparameter was estimated from the data (*kpar = "automatic"*). In the Recursive Feature Elimination process, feature importance was assessed based the variable importance output obtained from fitting a Random Forests classifier.

## Authors' contributions

YG participated in the design of the study, carried out data analysis, authored the R package ('*MVpower*') and was involved in the drafting of the manuscript. RB conceived the study, was involved in the study design, carried out the simulation studies and was involved in drafting the manuscript. AG was involved in the design of the study and in drafting the manuscript. RM was involved in study design and in drafting of the manuscript. All authors read and approved the final manuscript.

## Supplementary Material

Additional file 1 **Simulation Results - Supplement**. This file includes additional simulation results for the following settings: (i) Comparison of statistical power and average classification accuracy for classifiers KNN, PAM, RF and SVM, when *n *= 50; (ii) Estimates of average classification accuracy for classifiers KNN, PAM, RF and SVM, based on independent test sets. Click here for file
