# Supplementary material for: Sample size and statistical power considerations in high-dimensionality data settings: a comparative study of classification algorithms
Source: BMC Bioinformatics. 2010 Sep 3;11:447. doi: 10.1186/1471-2105-11-447 (PMC2942858; doi:10.1186/1471-2105-11-447)
Supplement: Additional file 1 — Simulation Results - Supplement. This file includes additional simulation results for the following settings: (i) Comparison of statistical power and average classification accuracy for classifiers KNN, PAM, RF and SVM, when n = 50; (ii) Estimates of average classification accuracy for classifiers KNN, PAM, RF and SVM, based on independent test sets. [file 1471-2105-11-447-S1.DOC]

**Simulation Results – Supplement**


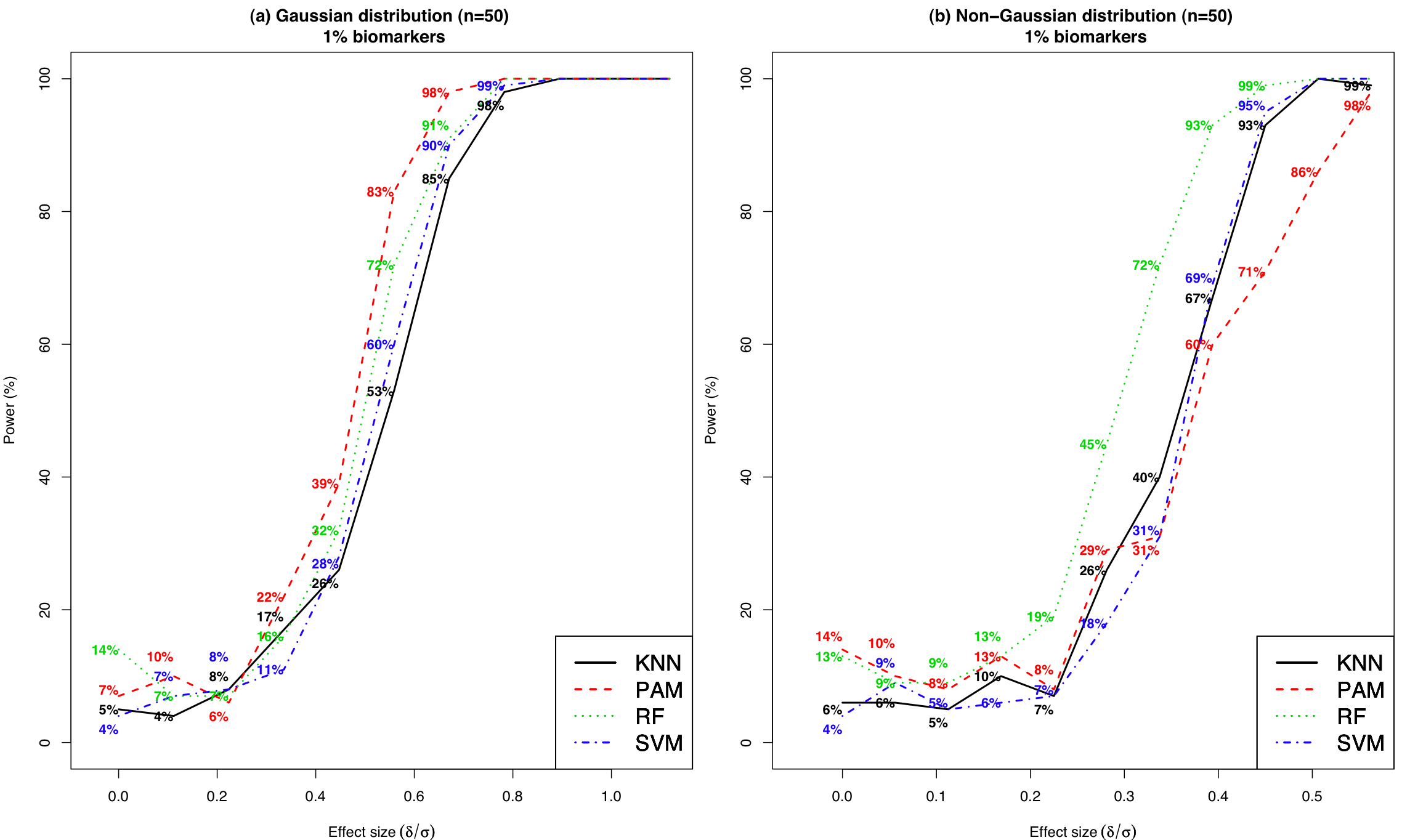


Figure S1: Comparison of statistical power of classifiers under the assumption of N=50 .

Each dataset included 1000 features per subject, where *n*=50 and *k*=1%. Results shown in Panel (a) were based on a Gaussian model for class conditional feature distributions. Results shown in Panel (b) were based on a mixture model to generate skewed, non-Gaussian class conditional feature distributions. Results are based on 100 simulated datasets.


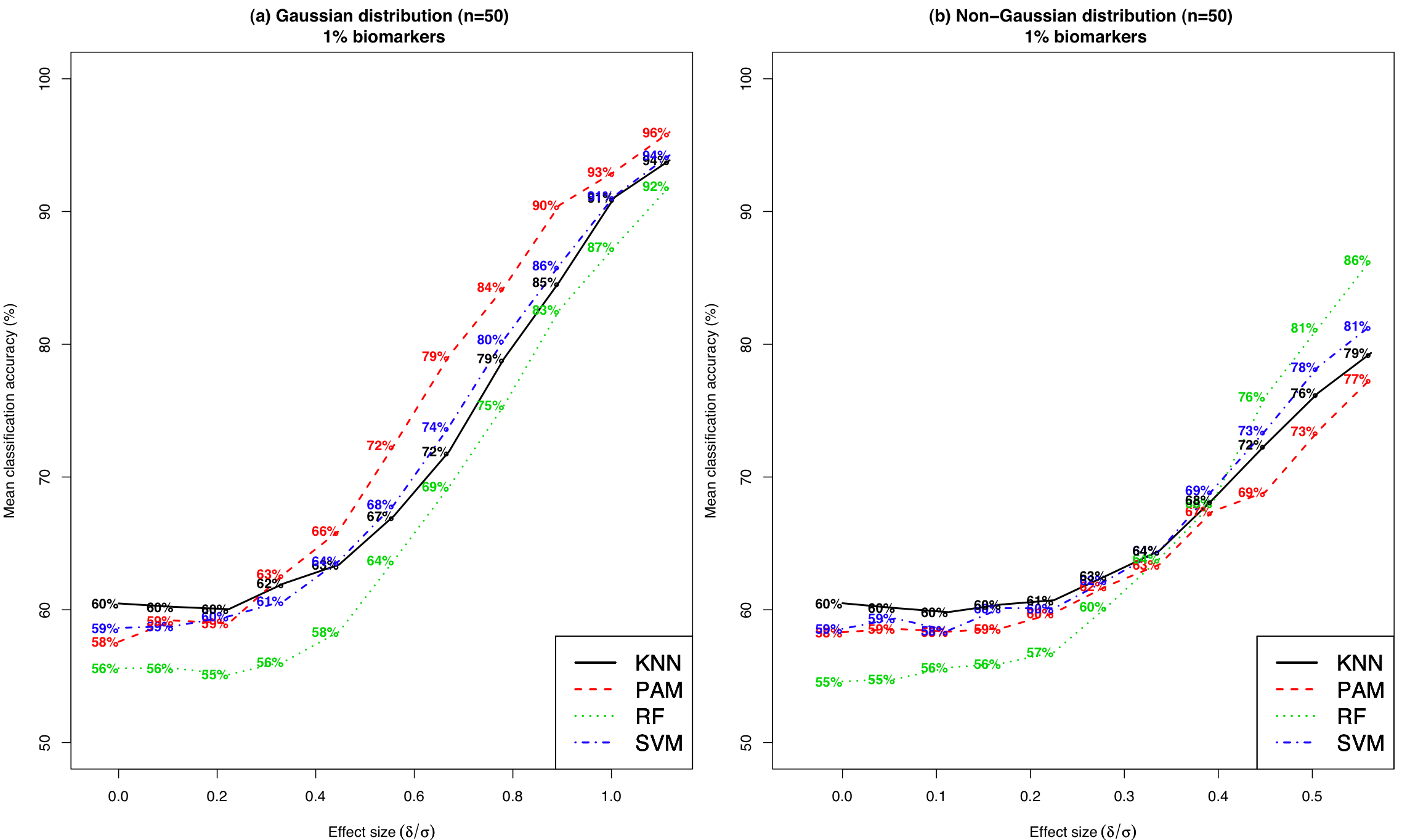


Figure S2: Comparison of average classification accuracy of classifiers under the assumption of N=50.

Each dataset included 1000 features per subject, where *n*=50 and *k*=1%. Results shown in Panel (a) were based on a Gaussian model for class conditional feature distributions. Results shown in Panel (b) were based on a mixture model to generate skewed, non-Gaussian class conditional feature distributions. Results are based on 100 simulated datasets.


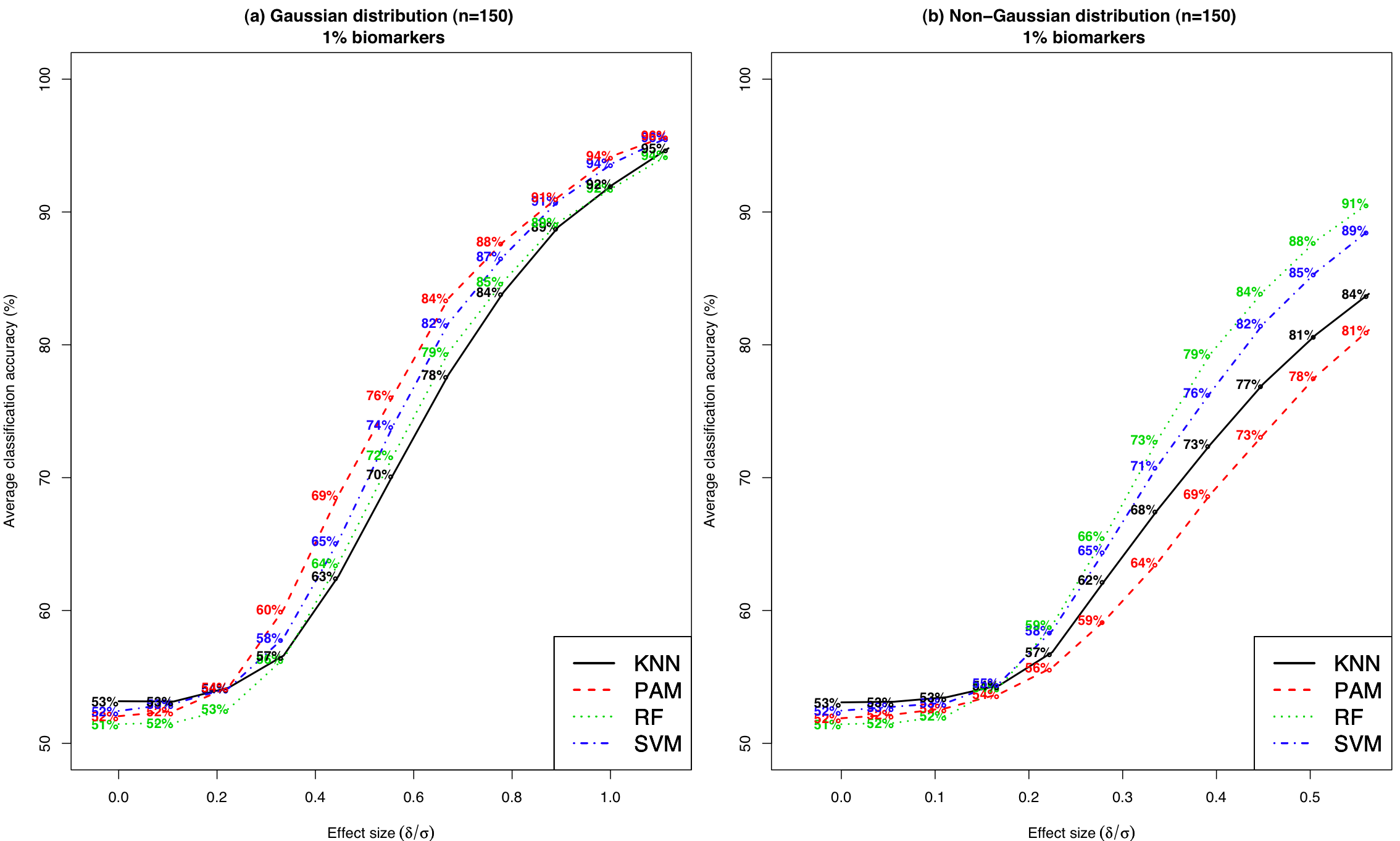


Figure S3: Comparison of average classification accuracy of classifiers. Estimates of average classification accuracy were based on simulated independent test datasets.

Each dataset included 1000 features per subject, where *n*=150 and *k*=1%. Results shown in Panel (a) were based on a Gaussian model for class conditional feature distributions. Results shown in Panel (b) were based on a mixture model to generate skewed, non-Gaussian class conditional feature distributions. Results are based on 100 simulated datasets. Average classification accuracy estimates were derived based on simulated independent test datasets of 400 subjects (*n*=200).
